# The Anti-Proliferative Effect of Boron Neutron Capture Therapy in a Prostate Cancer Xenograft Model

**DOI:** 10.1371/journal.pone.0136981

**Published:** 2015-09-01

**Authors:** Kiyoshi Takahara, Teruo Inamoto, Koichiro Minami, Yuki Yoshikawa, Tomoaki Takai, Naokazu Ibuki, Hajime Hirano, Hayahito Nomi, Shinji Kawabata, Satoshi Kiyama, Shin-Ichi Miyatake, Toshihiko Kuroiwa, Minoru Suzuki, Mitsunori Kirihata, Haruhito Azuma

**Affiliations:** 1 Department of Urology, Faculty of Medicine, Osaka Medical College, Osaka, Japan; 2 Department of Neurosurgery, Osaka Medical College, Osaka, Japan; 3 Radiation Oncology and Particle Radiation Oncology Research Center, Research Reactor Institute, Kyoto University, Sennan-gun, Osaka, Japan; 4 Research Center of Boron Neutron Capture Therapy, Research Organization for the 21st Century, Osaka Prefecture University, Sakai, Japan; University of Pécs Medical School, HUNGARY

## Abstract

**Purpose:**

Boron neutron capture therapy (BNCT) is a selective radiation treatment for tumors that preferentially accumulate drugs carrying the stable boron isotope, ^10^B. BNCT has been evaluated clinically as an alternative to conventional radiation therapy for the treatment of brain tumors, and more recently, recurrent advanced head and neck cancer. Here we investigated the effect of BNCT on prostate cancer (PCa) using an *in vivo* mouse xenograft model that we have developed.

**Materials and Methods:**

Mice bearing the xenotransplanted androgen-independent human PCa cell line, PC3, were divided into four groups: Group 1: untreated controls; Group 2: Boronophenylalanine (BPA); Group 3: neutron; Group 4: BPA-mediated BNCT. We compared xenograft growth among these groups, and the body weight and any motility disturbance were recorded. Immunohistochemical (IHC) studies of the proliferation marker, Ki-67, and TUNEL staining were performed 9 weeks after treatment.

**Results:**

The *in vivo* studies demonstrated that BPA-mediated BNCT significantly delayed tumor growth in comparison with the other groups, without any severe adverse events. There was a significant difference in the rate of freedom from gait abnormalities between the BPA-mediated BNCT group and the other groups. The IHC studies revealed that BNCT treatment significantly reduced the number of Ki-67-positive cells in comparison with the controls (mean±SD 6.9±1.5 vs 12.7±4.0, p<0.05), while there was no difference in the number of apoptotic cells, suggesting that BPA-mediated BNCT reduced PCa progression without affecting apoptosis at 9 weeks post-treatment.

**Conclusions:**

This study has provided the first preclinical proof-of-principle data to indicate that BPA-mediated BNCT reduces the *in vivo* growth of PCa. Although further studies will be necessary, BNCT might be a novel potential treatment for PCa.

## Introduction

Boron neutron capture therapy (BNCT) is a binary treatment modality for cancer that is based on accumulation of agents containing the nonradioactive isotope boron-10 (^10^B), a constituent of natural elemental boron, in cancer cells followed by irradiation with low-energy thermal neutrons to yield high linear energy-transfer alpha particles and recoiling lithium-7 nuclei [1, 2]. Because these particles have a short path length of 5–10 μm in water, the cytotoxic effects are confined within boron-10-containing cells, if ^10^B atoms are selectively accumulated in tumor cells. Thus, in order for BNCT to succeed, it requires selective delivery of large amounts of ^10^B to tumor cells.

^10^B-containing compounds can be accumulated selectively into tumor cells by several mechanisms. Two of the most common ^10^B-carriers used in clinical BNCT trials, designed for the treatment of malignant gliomas, melanomas, inoperable head and neck tumors, and oral cancer, are L-para-boronophenylalanine-^10^B (BPA, C9H1210BNO4) and sodium mercaptoundecahydrododecaborate-^10^B (BSH, Na210B12H11SH)[1]. BPA was originally evaluated as a boron delivery agent for melanoma, and BNCT of BPA-loaded tumors resulted in high levels of tumor control [3]. BPA is selectively and preferentially accumulated into tumor cells as a result of augmented amino acid metabolism by active transport across the cancer cell membrane in comparison with normal cells [4]. BPA has been utilized in experimental studies of brain tumor therapy [5, 6] following reports indicating that it was preferentially accumulated in rat gliosarcoma, human glioma xenografts, and murine mammary adenocarcinoma [7]. BSH relies on the blood-brain barrier to achieve selective accumulation in tumor cells relative to normal brain [8]. BSH does not cross the intact blood-brain barrier in the normal brain but is delivered to tumors because the tumor vasculature does not form the tight junctions associated with the blood-brain barrier. Since the boron concentrations achieved with BSH in the blood can be as high as in the tumor, damage to the vascular endothelial cells in the brain would be the primary determinant of radiation damage to the central nervous system [9].

BNCT has been evaluated clinically as an alternative to conventional radiation therapy for malignant brain tumors (gliomas), and more recently, recurrent locally advanced head and neck cancer [10]. However, there have been few reports on the use of BNCT for treatment of prostate cancer (PCa). In the present *in vivo* study, therefore, we assessed whether BPA-mediated BNCT would affect the growth of the xenografted androgen-independent PCa cell line, PC3. We found that BPA-mediated BNCT reduced the growth of PC3 xenografts without any severe adverse events.

This study provides the first preclinical proof-of-principle data to indicate that BPA-mediated BNCT reduces the growth of PCa xenografts *in vivo*, suggesting that this might be a promising new therapeutic approach for patients with PCa.

## Materials and Methods

### Cell lines

PC3 cells were purchased from the American Type Culture Collection (ATCC, Rockville, MD, USA). The cells were maintained in RPMI1640 supplemented with 10% fetal bovine serum (FBS) and 1% penicillin/streptomycin (Life Technologies, Burlington, Ontario, Canada) at 37°C in a 5% CO_2_ atmosphere.

### Boron compounds

BPA was kindly provided by Dr. Mitsunori Kirihata (Research Center for Boron Neutron Capture Therapy, Research Organization for the 21st Century, Osaka Prefecture University, Sakai, Japan), and converted to a fructose complex. An aqueous solution of the BPA complex was prepared at a concentration of 250 mg/ml (21.28 mg ^10^B/ml). In order to evaluate ^10^B concentrations in mice, BPA was injected intraperitoneally at a dose of 250 mg/kg in accordance with previous studies [11, 12].

### Determination of boron concentration by ICP-AES

This study was carried out in strict accordance with the recommendations in the Guide for the Care and Use of Laboratory Animals of the National Institutes of Health (S1 ARRIVE Checklist). The protocol was approved by the Osaka Medical College Animal Care and Use Committee (Permit Number: 26075). All surgeries were performed under anesthesia by 2% isoflurane inhalation, and all efforts were made to minimize suffering. Quantitative determination of boron was carried out by the inductively coupled plasma atomic emission spectrometric (ICP-AES) method. PC3 cells (1x10^6^) with 50 μl of Matrigel (Becton Dickinson Labware, Franklin Lakes, NJ) and 50 μl of serum-free RPMI1640 were injected subcutaneously into the back of the leg of 6~8-week-old male athymic nude mice with a 27-gauge needle under anesthesia by 2% isoflurane inhalation. At week 2 after PC3 cell injection, when PC3 xenografts were palpable, BPA complex at a concentration of 250 mg/ml (21.28 mg ^10^B/ml) was injected intraperitoneally into tumor-bearing mice. Samples of heart, blood, brain, liver, kidney, lung, and tumor were collected at 2 and 4 h after injection, digested with nitric acid solution, stored in distilled water at room temperature overnight, and then subjected to the boron concentration assay using an ICP-AES instrument (SPS 3100;SSI; Nanotechnology, Tokyo, Japan). The numbers of mice in each group were 3.

### BNCT for PC3 xenografts

PC3 cells (1x10^6^) with 50 μl of Matrigel and 50 μl of serum-free RPMI1640 were injected subcutaneously into the back of the leg of 6~8-week-old male athymic nude mice with a 27-gauge needle under anesthesia by 2% isoflurane inhalation. The day of injection of PC3 cells was taken as the starting point and defined as day 0. At week 2 after PC3 cell injection, when PC3 xenografts were palpable, the mice were divided into four groups: Group 1: untreated control, Group 2: BPA, Group 3: neutron, and Group 4: BPA-mediated BNCT. The numbers of mice in each group were 6. In Groups 3 and 4, the tumors in the leg were subjected to thermal neutron beam irradiation at the heavy water facility of Kyoto University Research Reactor for 60 min at a power of 1 MW. Each mouse was held within a specially designed acrylic cage during irradiation, and a LiF plate (50 mm thick) was used to shield the body from thermal neutrons while the tumor-inoculated leg was exposed. In Group 4, BPA was administered intraperitoneally 2 hours before irradiation at a dose of 250 mg/kg. Tumor volume measurements were performed once a week and calculated using the formula: length x width x depth x 0.5236 [13]. At 9 weeks, the mice were sacrificed and the tumors were prepared for histological examination.

### Histological examination

For immunohistochemistry, tissues were embedded in OCT compound (Miles Scientific, Elkhardt, IN) and snap-frozen in liquid nitrogen. Frozen sections 6 μm thick were mounted on silane-coated glass slides, and air-dried for 1 h. Cell apoptosis was confirmed by detection of fragmented DNA, using a DeadEnd Colorimetric TUNEL System (Promega, Madison, WI) in accordance with the manufacturer’s instructions. As markers of cell proliferation, sections were stained with anti-Human Ki-67 eFluor 570 (eBioscience, Frankfurt, Germany, 1:200) and anti-SMa-actin (Sigma-Aldrich Japan K.K., Tokyo, Japan, 1:500). Nuclear counter-staining was performed by incubation with 4',6-diamidino-2-phenylindole (DAPI) solution (Sigma-Aldrich, 1 μg/ml in PBS) for 10 min at room temperature. Double-positive cells were counted and averaged for quantitative analysis.

### Statistical analysis

All values obtained in immunohistochemical (IHC) studies are presented as mean ± SD, and those for *in vivo* body weight, tumor volume, and measurement of ^10^B concentrations are presented as mean ± SEM. Statistical comparisons between two groups were performed by unpaired Student’s *t* test. The periods during which mice remained free of any gait abnormalities were converted to Kaplan-Meier plots, and the significance of differences between them at p<0.05 was calculated using the generalized log-rank test.

## Results

### Timing of peak ^10^B concentration after intraperitoneal injection of BPA

**Fig 1:**
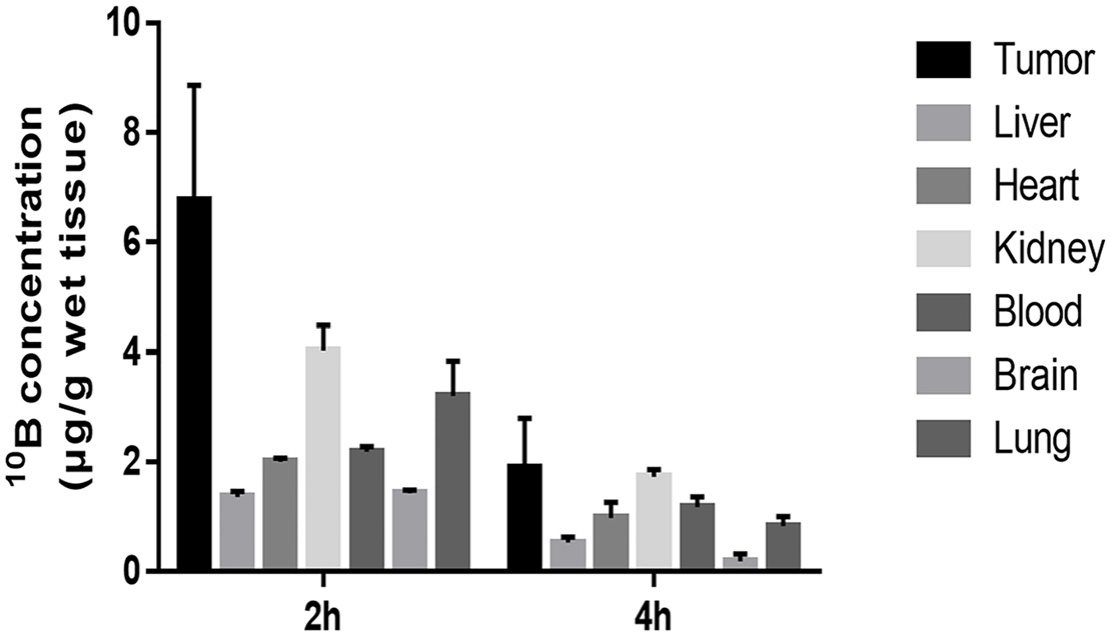
Time course changes of ^10^B concentration in 6 tissues. ^10^B concentration in the heart, blood, brain, liver, kidney, lungs, and tumor of PC3 tumor-bearing mice were measured by ICP-AES instrument at 2 and 4 hour after intraperitoneal injection of 250 mg/kg BPA.

^10^B concentrations were first measured by the boron concentration assay using an ICP-AES instrument in the heart, blood, brain, liver, kidney, lungs, and tumor of PC3 tumor-bearing mice in order to assess the optimum period for neutron capture after intraperitoneal injection of BPA at 250 mg/kg with a 27-gauge needle under anesthesia by 2% isoflurane inhalation. In every organ, mice showed high peak ^10^B concentrations at 2 hours, as compared with those at 4 hours. ^10^B concentration in tumor both at 2 and 4 hours were high as compared with ^10^B concentration in other organs, especially at 2 hours (^10^B concentrations in tumor at 2 and 4 hours were 6.77±2.09 and 1.89±0.91 μg/g, respectively) (Fig 1). Therefore we concluded that the best time point for neutron capture therapy was 2 hours after injection.

### Assessment of effect of BPA-mediated BNCT on body weight of mice during the treatment period

**Fig 2:**
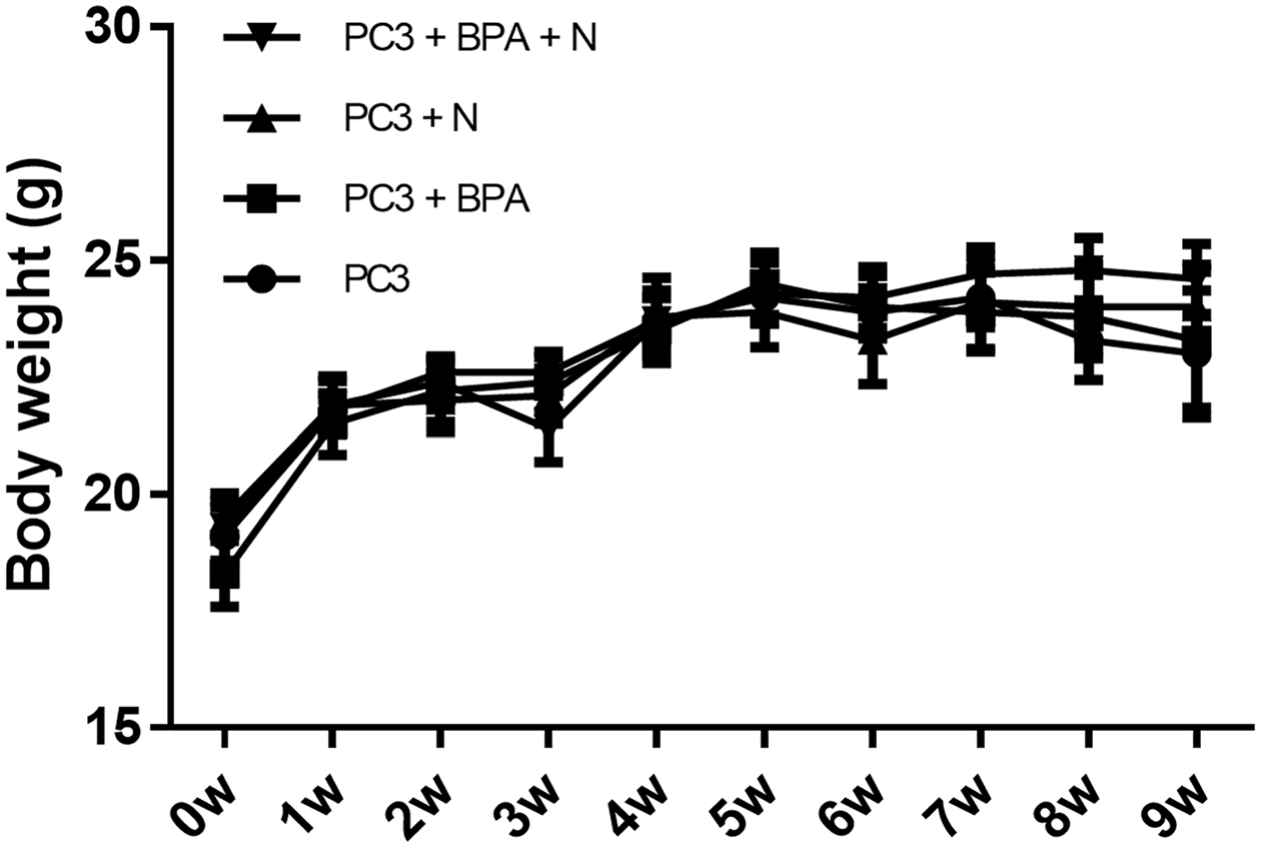
The mean body weight of mice in four groups. Mean body weight of mice (Group 1: untreated control, Group 2: BPA, Group 3: neutron, Group 4: BPA-mediated BNCT) from 0 to 9 week were shown. Each point represents the mean body weight ± SEM.

In order to assess whether BPA-mediated BNCT influenced the growth and body weight of mice, the animals were monitored once a week from 0 week to 9 weeks. The day of injection of PC3 cells was taken as the starting point and defined as day 0. At week 2 after PC3 cell injection, when PC3 xenografts were palpable, the mice were divided into four groups: Group 1: untreated control, Group 2: BPA, Group 3: neutron, and Group 4: BPA-mediated BNCT. The mean body weights of the mice in the four groups are shown in Fig 2. No significant effect on animal body weight was observed during the treatment period. These results indicated that none of the treatments—BPA, neutron irradiation or BPA-mediated BNCT—produced severe side effects in this *in vivo* mouse xenograft model.

### Effect of BPA-mediated BNCT treatment on growth of PC3 xenografts

**Fig 3:**
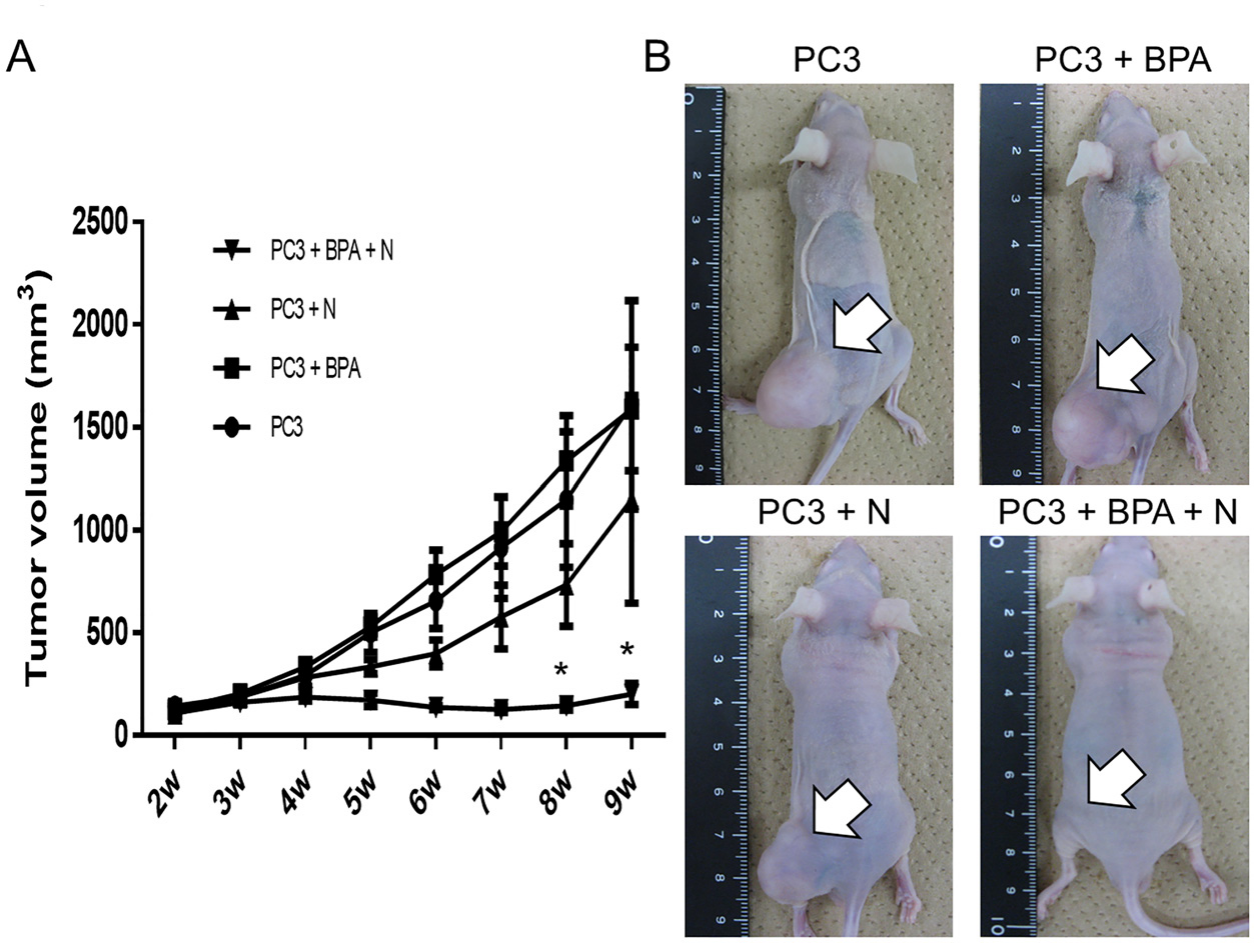
**(A) Tumor volume of mice in four groups 2 to 9 weeks after PC3 injection.** Mean tumor volume of mice (Group 1: untreated control, Group 2: BPA, Group 3: neutron, Group 4: BPA-mediated BNCT) from 2 to 9 week were shown. Each point represents the mean tumor volume ± SEM. *P<0.05 differs from Group 1, 2, and 3 by Student’s t test. **(B) Macroscopic findings in four groups at 9 weeks.** White arrows indicate sites of tumor.

The sizes of the tumors in the four groups of model mice were monitored for up to 9 weeks. Untreated control mice (Group 1) and BPA-treated mice (Group 2) showed rapid xenograft growth, and the average tumor volume at 9 weeks after the start of the experiment was 1611 mm^3^ and 1589 mm^3^, respectively. Mice subjected to neutron irradiation (Group 3) showed slightly slow xenograft progression, and the average tumor volume reached 1151 mm^3^ at 9 weeks. However, when the xenografts were subjected to BPA-mediated BNCT (Group 4), tumor progression was markedly reduced from 2 weeks after BNCT, and average tumor volume at 9 weeks was 202 mm^3^. The differences in tumor volume between Group 4 and Groups 1, 2 and 3 at 8 and 9 weeks after the start of the experiment were significant (Fig 3A) (p<0.05). Macroscopic observation revealed that mice in Groups 1, 2 and 3 had clearly enlarged palpable tumors, whereas the tumors in Group 4 mice were almost undetectable (Fig 3B). These xenograft studies demonstrated that BPA-mediated BNCT significantly delayed PCa growth without any severe adverse events.

### Effect of BPA-mediated BNCT in terms of gait abnormalities

**Fig 4:**
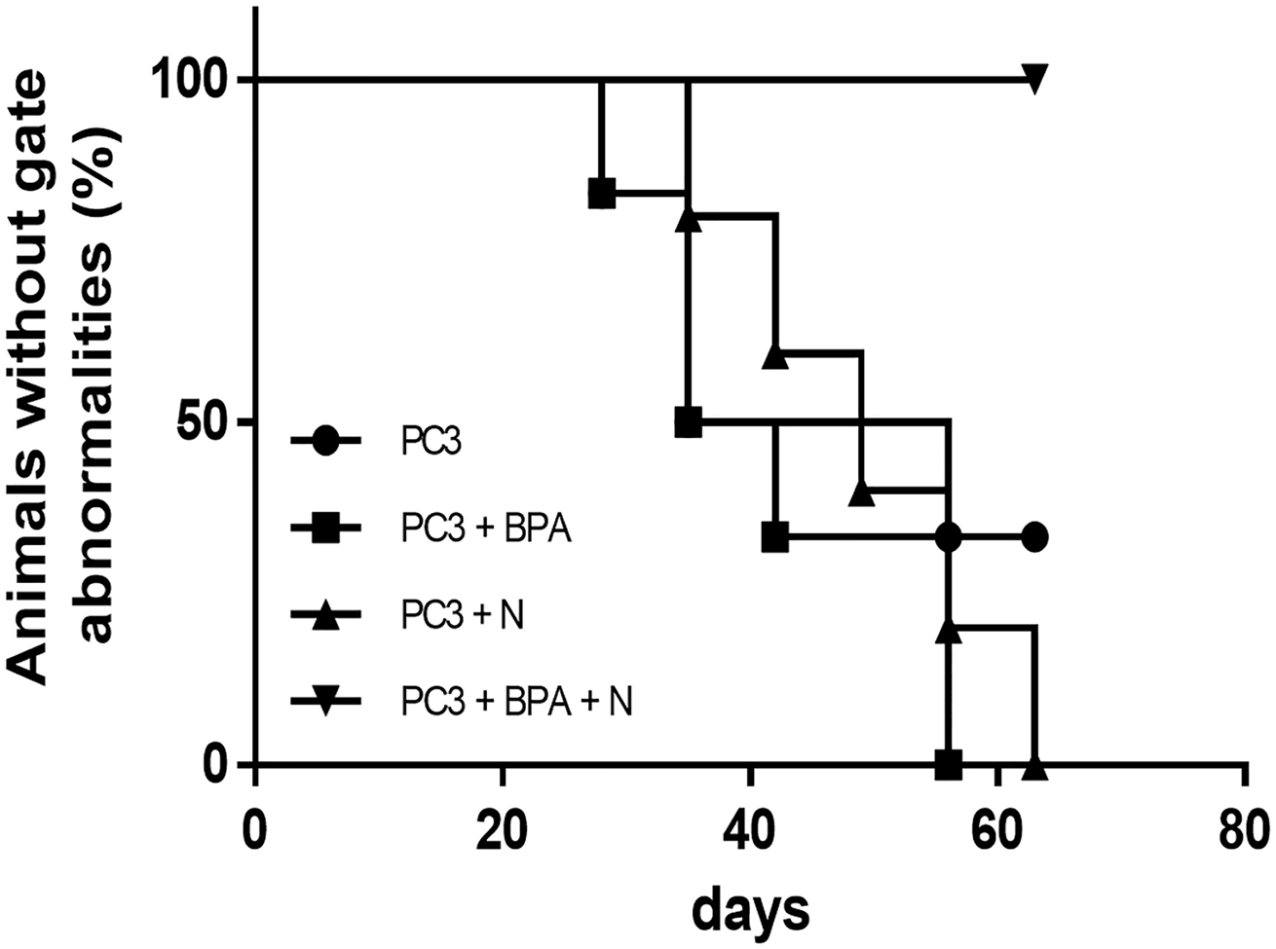
Percentages of animals without gait abnormalities in the four groups. Gait abnormalities of mice (Group 1: untreated control, Group 2: BPA, Group 3: neutron, Group 4: BPA-mediated BNCT) were observed. The percentages of animals without gait abnormalities differed significantly between Group 4 and Groups 1, 2 and 3 (p<0.05).

In order to examine how much the degree to which PC3 tumor xenografts influenced the gait of freely moving mice, we next assessed the motility of mice in all groups. Mice that had been treated with BPA (Group 2) and neutron irradiation (Group 3) developed gait abnormalities by day 56 and day 63, respectively, whereas about 66.7% of the untreated control mice (Group 1) also showed gait anomalies by day 56. However, none of the mice subjected to BPA-mediated BNCT (Group 4) developed motility disturbance during the treatment period (Fig 4). Consistent with the result that the differences in tumor volume between Group 4 and Groups 1, 2 and 3 at late stage during the treatment were significant, the rates of freedom from gait abnormalities differed significantly between Group 4 and Groups 1, 2 and 3 (p<0.05). These results demonstrated that PC3 xenograft growth was markedly suppressed by BPA-mediated BNCT.

### Immunohistochemical (IHC) studies of xenograft tissue

**Fig 5:**
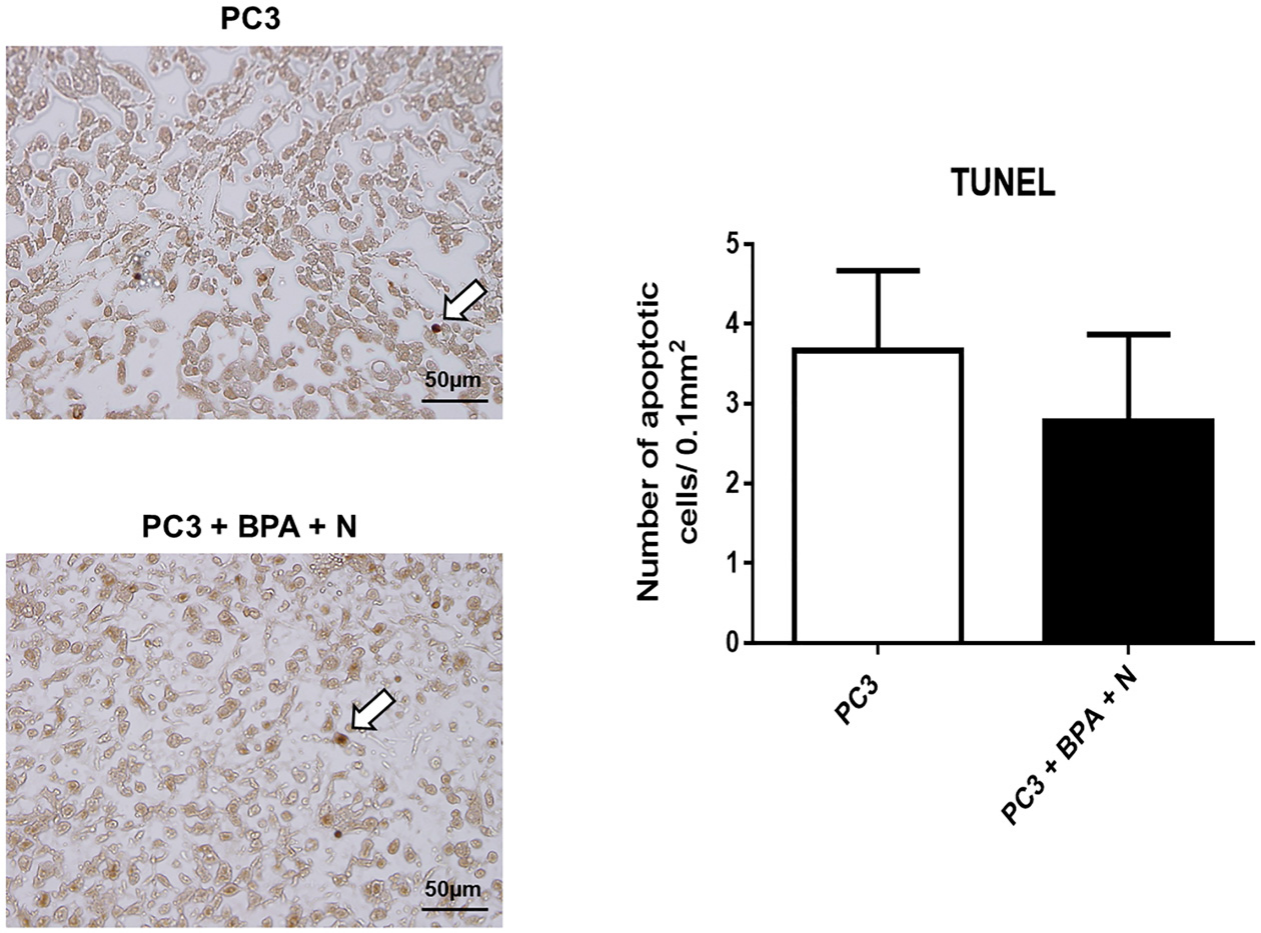
TUNEL staining on PC3 xenograft specimens. Xenograft tissues of mice (Group 1: untreated control and Group 4: BPA-mediated BNCT) were stained with TUNEL and the number of apoptotic cells was quantified. White arrows indicate TUNEL+ cells.

**Fig 6:**
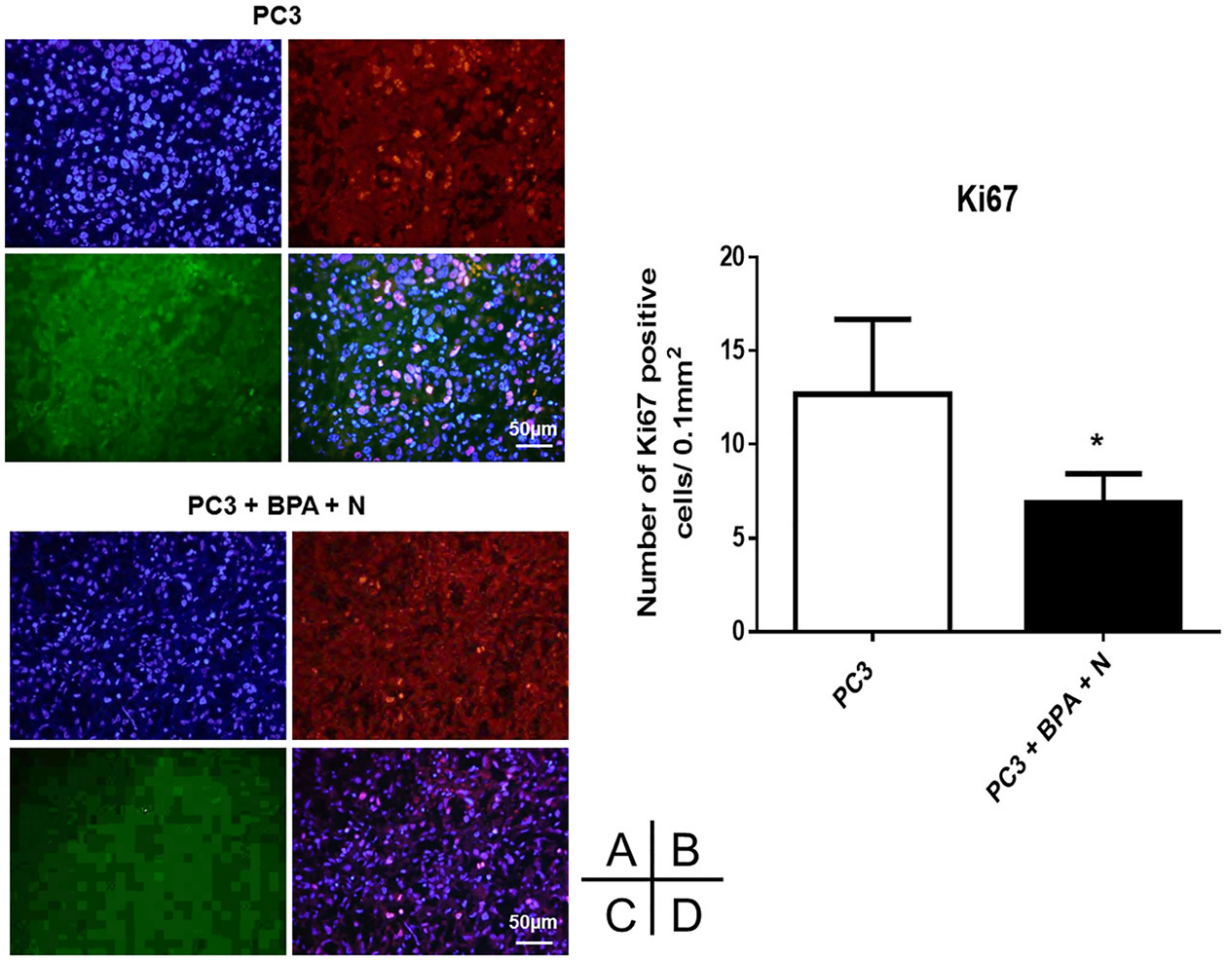
Ki67 staining on PC3 xenograft specimens. Xenograft tissues of mice (Group 1: untreated control and Group 4: BPA-mediated BNCT) were stained with Ki67, SMa-actin, and DAPI (A:DAPI B:Ki67 C:SMa-actin D:Mixed). Quantification of Ki67-positive cells was shown.

At 9 weeks post-treatment, tissue samples from untreated control mice (Group 1) and mice subjected to BPA-mediated BNCT (Group 4) were first examined histologically using standard hematoxylin and eosin (H&E) staining after formalin fixation. In both groups, vacuolated tumor cells with multiple nuclear fragmentations were observed, although the histological findings did not differ significantly between them (data not shown). Next, in order to assess how BPA-mediated BNCT affected PC3 xenograft growth, immunohistochemistry for the proliferation marker, Ki-67, and TUNEL staining were performed on PC3 xenograft specimens from Groups 1 and 4. Apoptosis was observed in both groups, without any obvious differences between them, and quantification of apoptotic cells also indicated no significant inter-group difference (Fig 5). However, double-positive staining for Ki-67 was observed significantly more frequently in Group 1 than in Group 4 (mean±SD 12.7±4.0 vs 6.9±1.5, respectively, p<0.05) (Fig 6). These IHC studies suggested that BPA-mediated BNCT slowed PCa progression without affecting apoptosis at 9 weeks post-treatment.

## Discussion

PCa is now a major and escalating international health problem among men, and is one of the most common malignant solid tumors in Western countries [14]. Androgen deprivation therapy (ADT) is the gold standard for recurrent or advanced PCa [15]. Although most PCa patients are initially dependent on androgens for tumor growth, and apparently respond well to ADT, most patients invariably develop treatment resistance at some stage, developing a castration-resistant (CR) status with an eventual fatal outcome even after potentially curative treatment. Therefore, there is an urgent need for a novel therapeutic strategy that can overcome the emergence of CR PCa.

BNCT is a selective tumor cell radiation treatment for tumor cells that is based on preferential intracellular accumulation of drugs carrying the stable boron isotope, ^10^B. The first BNCT trials were conducted on ten patients with terminal glioblastoma multiforme brain tumors between 1951 and 1953 [16]. In this trial, water-soluble ^10^B-enriched sodium borate (borax, Na_2_B_4_O_7_), which was considered relatively non-toxic at therapeutic concentrations of up to 200 mg per kg body weight, was used as the initial BNCT drug. However, all ten patients died due to tumor recurrence without prolongation of their survival times. Further clinical trials were then conducted with several ^10^B-containing compounds, and BNCT was introduced to Japan in 1968 by the neurosurgeon Hiroshi Hatanaka [17], who used sodium borocaptate (BSH) and a low-energy thermal neutron beam with low tissue-penetrating properties. In more recent clinical trials in Japan, our co-authors Miyatake and Kawabata have initiated several protocols employing a combination of BPA (500 mg/kg) and BSH (100 mg/kg), infused i.v. over 2 hours, followed by neutron irradiation at Kyoto University Research Reactor Institute (KURRI) [18, 19]. Miyatake et al. demonstrated that BNCT conferred a survival benefit for patients with recurrent malignant glioma, especially for those who were at high risk [19]. Kawabata et al. reported that BNCT utilizing sodium borocaptate and boronophenylalanine simultaneously in combination with X-irradiation enhanced the survival of patients with newly diagnosed glioblastoma, in comparison with BNCT alone [18]. Another study by that group demonstrated that a combination of transferrin-conjugated polyethylene glycol (TF-PEG) liposomes encapsulating sodium borocaptate and Iomeprol with intratumoral convection-enhanced delivery (CED) enabled not only precise and potent targeting of boron to the tumor tissue, but also tracing of boron administered intratumorally using real-time computed tomography [20].

For extracranial tumors, BNCT was reported to be a new and promising treatment approach in 26 patients (19 squamous cell carcinomas (SCC), 4 salivary gland carcinomas and 3 sarcomas) with recurrent and far advanced head and neck malignancies [21]. Recently, one of our co-authors, Suzuki, and colleagues reviewed the outcomes of BNCT in 62 patients with either locally recurrent or newly diagnosed unresectable head or neck cancers [22]. Using either BPA alone, or BPA combined with other boron compounds, the dose constraint was delivery of <10–12 Gy-eq to the skin or oral mucosa, and this was shown to be a feasible dose-estimation method for BNCT in this setting.

Moreover, in order to identify potential tumors that may be amenable to BNCT and to improve treatment plans prior to BNCT, the accumulation of BPA to the tumor and surrounding normal tissue is imaged and quantified by an ^18^F-BPA PET study before BNCT [23, 24]. Thermal neutron fluence is measured by radioactivation of gold wires (0.25 mm in diameter and 1.0 cm long) placed on the skin surface of the lesion, and the dose-volume histogram (DVH) parameters are evaluated by a Simulation Environment for Radiotherapy Applications (SERA) system and the Japan Atomic Energy Research Institute’s Computational Dosimetry System, which are currently available BNCT treatment-planning systems [25].

On the other hand, there have been few reports of clinical trials of BNCT or basic research in the context of PCa. Schinazi et al. initially indicated that among the carboranyl nucleotides beta-D-5-o-carboranyl-2'-deoxyuridine (D-CDU), 1-(beta-L-arabinosyl)-5-o-carboranyluracil (D-ribo-CU) and the nucleotide base 5-o-carboranyluracil (CU), CU was the most suitable compound for BNCT *in vivo* using the androgen-dependent PCa cell line, LNCaP [26]. In another study, enhanced cell killing was observed when the BPA-loaded PCa cell line, DU145, was irradiated using a modified enhanced thermal neutron beam (METNB) assembly developed at Fermi National Accelerator Laboratory (Fermilab) [27]. Recently, Gifford et al. demonstrated that liposome-based delivery of a boron-containing cholesteryl ester compound (BCH) was capable of introducing sufficient boron into PC3 cells for BNCT, and that high-linear energy transfer (High-LET) particles and ^7^Li nuclei generated by ^10^B thermal neutron capture significantly decreased the colony formation ability of targeted PC3 cells *in vitro* [28].

In the present study, we carried out *in vivo* experiments using BPA-mediated BNCT in the androgen-independent PCa cell line, PC3. Our xenograft studies demonstrated that BPA-mediated BNCT significantly delayed PCa growth with no severe adverse events as compared with other groups (untreated control, BPA only, and neutron irradiation only), and IHC studies suggested that BPA-mediated BNCT reduced PCa progression without affecting apoptosis. However, before clinical trials of BNCT for PCa patients can begin, some problems remain to be resolved. One of them is neutron capture, as neutrons can only reach organs close to the body surface, and their effect decreases with increasing depth. For this reason, if the prostate is neutron-irradiated from a normal direction, BNCT would probably not be effective even if BPA is successfully delivered to PCa. Therefore, we consider that if neutrons could be delivered transperineally from a normal direction, then the distance between the prostate and the body surface would be considerably reduced.

With regard the toxicity after BNCT, we note that we have previously treated 62 patients with head-neck cancer, and observed hyperamylasemia (38.6%), fatigue (6.5%), mucositis/stomatitis (9.7%) and pain (9.7%), as major acute Grade 3 or 4 toxicities, while all of which were manageable [22]. BNCT for PCa patients has not been performed clinically, however, the above-mentioned side effects would be observed if neutrons could be delivered transperineally.

In conclusion, our present *in vivo* study has provided the first preclinical proof-of-principle data to indicate that BPA-mediated BNCT can suppress the growth of androgen-independent PC3 xenografts. In order for BNCT to be applied to PCa patients in clinical trials, however, some problems such as the direction of neutron irradiation need to be resolved. However, BNCT appears to hold promise as a novel treatment for PCa.

## Supporting Information

S1 ARRIVE Checklist NC3Rs ARRIVE Guidelines Checklist. (RTF)
